# Supplementary material for: Repurposing antiviral agents against mucormycosis
Source: PLoS One. 2026 Feb 18;21(2):e0342559. doi: 10.1371/journal.pone.0342559 (PMC12915971; doi:10.1371/journal.pone.0342559)
Supplement: S1 Table — (DOCX) [file pone.0342559.s001.docx]

# Supplementary Information

**Supplementary Table 1: Description of hits from screening of antiviral compound library**

| **Compound** | **Description** | **Percentage Growth Inhibition** |
| --- | --- | --- |
| Tunicamycin | Antibiotic | 87.41 |
| BLT-1 | SR-B1 inhibitor | 84.85 |
| MSC109 | Cancer study agent | 88.18 |
| Tubacin | Cancer study agent | 71.31 |
| IMB-301 | HIV-1 inhibitor | 87.49 |
| Carmofur | Anticancer agent | 73.71 |
| NH125 | Research agent with antibacterial properties | 88.77 |
| U18666A | Cholesterol transport inhibitor | 87.15 |
| Punicalagin | Polyphenolic compound found in pomegranate | 70.9 |
| Hinokitiol | Natural compound derived from trees | 83.22 |
| Cetylpyridinium | Antiseptic in oral care | 88.23 |
| Baicalein | Flavonoid derived from *Scutellaria baicalensis* | 71.28 |
| Octylgallate | Food preservative | 70.3 |
| Theaflavin | Polyphenol derived from tea | 76.3 |
| Obefazimod | Investigational drug for ulcerative colitis and HIV | 83.32 |
| Chebulagic Acid | Polyphenolic compound from Teminalia chebula | 77.42 |
| Corilagin | Ellagitannin found in medicinal plants | 73.37 |
