# Supplementary material for: Repurposing antiviral agents against mucormycosis
Source: PLoS One. 2026 Feb 18;21(2):e0342559. doi: 10.1371/journal.pone.0342559 (PMC12915971; doi:10.1371/journal.pone.0342559)
Supplement: Attachment — Submitted filename: Response to Reviewers.docx [file pone.0342559.s004.docx]

**Dear Dr. Ahmad,**

We thank you and the reviewers for the time and effort dedicated to evaluating our manuscript, *“Repurposing Antiviral Agents Against Mucormycosis.”* We appreciate the comments, which have strengthened the scientific quality and clarity of our work.

We have revised the manuscript thoroughly in response to all reviewer and editorial suggestions. A detailed, point-by-point Response to Reviewers document has been prepared, addressing each concern individually. All changes made in the manuscript are clearly indicated in the file labeled “Revised Manuscript with Track Changes.” A clean version without tracked changes has also been provided.

We believe these revisions significantly enhance the manuscript and address all concerns raised by the reviewers.

We appreciate the opportunity to revise our work and hope that the improved manuscript is now suitable for publication in PLOS ONE.

Sincerely,

Ammar A. Khan

**Editor comments:**

**1. Please ensure that your manuscript meets PLOS ONE's style requirements, including those for file naming. The PLOS ONE style templates can be found at**

[**https://journals.plos.org/plosone/s/file?id=wjVg/PLOSOne_formatting_sample_main_body.pdf**](https://journals.plos.org/plosone/s/file?id=wjVg/PLOSOne_formatting_sample_main_body.pdf) **and**

[**https://journals.plos.org/plosone/s/file?id=ba62/PLOSOne_formatting_sample_title_authors_affiliations.pdf**](https://nam04.safelinks.protection.outlook.com/?url=https%3A%2F%2Ftrack.editorialmanager.com%2FCL0%2Fhttps%3A%252F%252Fjournals.plos.org%252Fplosone%252Fs%252Ffile%253Fid%3Dba62%252FPLOSOne_formatting_sample_title_authors_affiliations.pdf%2F1%2F010f01991876c1f3-50692caf-1626-48a3-902e-cad7b36510a7-000000%2F6zb6ZWocVcBurJtDwitV_DdtColLnek3bZPT3DY6PYs%3D227&data=05%7C02%7Cammarakhan%40vt.edu%7C02d7e075277549106aa908ddec636a96%7C6095688410ad40fa863d4f32c1e3a37a%7C0%7C0%7C638926633915811185%7CUnknown%7CTWFpbGZsb3d8eyJFbXB0eU1hcGkiOnRydWUsIlYiOiIwLjAuMDAwMCIsIlAiOiJXaW4zMiIsIkFOIjoiTWFpbCIsIldUIjoyfQ%3D%3D%7C0%7C%7C%7C&sdata=b2vriC0Y7OKFeGCYlYeAuDDYirERmBfxff%2BNxqZjJmY%3D&reserved=0)

**2. Thank you for stating the following financial disclosure:
“This work was supported by the National Institute of Health Grant R01AI141439.”**

**Please state what role the funders took in the study. If the funders had no role, please state: "The funders had no role in study design, data collection and analysis, decision to publish, or preparation of the manuscript."
If this statement is not correct you must amend it as needed.
Please include this amended Role of Funder statement in your cover letter; we will change the online submission form on your behalf.**

**3. Thank you for stating the following in your Competing Interests section:
“None”**

**Please complete your Competing Interests on the online submission form to state any Competing Interests. If you have no competing interests, please state "The authors have declared that no competing interests exist.", as detailed online in our guide for authors at** [**http://journals.plos.org/plosone/s/submit-now**](http://journals.plos.org/plosone/s/submit-now) **This information should be included in your cover letter; we will change the online submission form on your behalf.**

**4. Please ensure that you refer to Figure 2 in your text as, if accepted, production will need this reference to link the reader to the figure.**

**5. Please include captions for your Supporting Information files at the end of your manuscript, and update any in-text citations to match accordingly. Please see our Supporting Information guidelines for more information:** [**http://journals.plos.org/plosone/s/supporting-information**](http://journals.plos.org/plosone/s/supporting-information)**.**

**6. If the reviewer comments include a recommendation to cite specific previously published works, please review and evaluate these publications to determine whether they are relevant and should be cited. There is no requirement to cite these works unless the editor has indicated otherwise.**

**REVIEWER 1 COMMENTS:**

**Query 1.**

Please upload Institutional Ethics Committee clearance report.

**Response 1.**

The use of *Rhizopus delemar* 99-880 and other fungal strains in this study was covered under our Institutional Biosafety Committee (IBC) approval (IBC Protocol #20-039; Virginia Tech).

**Query 2.**

How many times were each experiment repeated?

**Response 2.**

Details for each experiment are as follows: Screening was performed with the hits from the screening being confirmed through a subsequent confirmation screen. Evaluation of the potent hits was done in three independent experiments and MICs reported are the mode value after being observed visually. Checkerboard assay was done in three individual experiments using individual cultures and MIC values reported are the mode value. Growth kinetics and evaluation of cytotoxicity against vero cell line was done in two individual experiments.

**Query 3.**

Screening for antifungal activity in Figure 1 was done to a final time of 24 hours while time kill assays were read up to 48 hours. Why this discrepancy.

**Response 3.**

As per the CLSI M38A2 Protocol, incubation time for *Rhizopus* is 24 hours prior to reading MICs. For time kill assay we extended the duration, so we could assess the effect of the treatments on the growth over a longer period.

**Query 4.**

Please label Figure 1 A and Figure 1 B better to make them self explanatory.

**Response 4.**

Figures have been labelled accordingly.

**Query 5**

Heading of Figure 1 refers to the fungi as Mucor - it should be Mucorales - also note were these experiments not conducted on any non-Mucorales like Scedosporium

**Response 5.**

The heading for figure 1 has been rectified. In-vitro antifungal activity for both the standalone hits and the combination hits were tested against *Scedosporium* species as shown in Table 1 and Table 2.

**Query 6.**

**Was any hormetic effect ever detected in any of the time kill assays?**

**Response 6.**

No hormetic effect was observed for the compounds tested at the concentrations tested.

**Query 7.**

**Mention limitations of your study at the end of Discussion.**

**Response 7.**

Limitations have been added in the discussion section. See Pg. 8 - Lines 383-389 in the file Revised Manuscript.

**REVIEWER 2 COMMENTS:**

**Query 1.**

The manuscript entitled "Repurposing Antiviral Agents Against Mucormycosis" by Khan et al. represents a follow up study of several previous studies published under references no. [17-23] by the same group. They highlight the efficacy of HIV protease inhibitors against invasive fungal infections, including candidiasis and cryptococcosis, and the potential of antivirals in antifungal therapy. The authors screened a library of a total of 618 antiviral compounds against
Rhizopus delemar in the presence of a subinhibitory concentration of amphotericin B (0.0625 μg/mL) and performed microdilution to determine the minimal inhibitory concentrations for antifungal activity of the four most potent hits, IMB-301, BLT-1, U18666A, and obefazimod against a panel of 14 fungal strains (Mucorales, n=8) by microdilution. Cytotoxicity was measured on kidney cells. The compounds selected appeared to be highly effective against Rhizopus in in vitro antifungal activity testing, whilst cytotoxicity is low. However, information on the mode of action is missing.
Mucorales like Rhizopus are known to evade the immune system. Spores could survive within professional phagocytes like macrophages and monocytes and escape from antifungal accessibility.

How does administration of these compounds alter the intracellular fungal clearance of Rhizopus in professional phagocytes. A killing assay displaying the intracellular accessibility of these antiviral drugs within macrophages is highly recommended.

**Response 1.**

**Fig.1**. Intracellular activity of U18666A and IMB-301, alongside amphotericin B and posaconazole as positive controls.

An intracellular killing assay was performed using the J774 murine macrophage cell line to evaluate the ability of antiviral compounds to enhance clearance of *Rhizopus delemar* 99-880 within professional phagocytes. Macrophages were seeded into 96-well tissue-culture–treated plates and infected with *R. delemar* spores at a multiplicity of infection (MOI) of 1:10. Following a 3-hour incubation period to allow phagocytosis, wells were washed thoroughly with warm PBS to remove non-internalized conidia. Infected macrophages were then exposed to test compounds for 24 hours.

After drug treatment, macrophages were lysed with 0.1% Triton X-100 to release intracellular spores. Lysates were serially diluted in PBS, and 50 µL aliquots of each dilution were plated onto potato dextrose agar (PDA). Plates were incubated at 37 °C for 24–48 hours, and viable fungal burden was quantified by colony enumeration. Intracellular killing was expressed as CFU/mL relative to untreated infected controls. At the used concentrations, none of the treatments reduced intracellular fungal counts comparative to untreated control, however Amphotericin B demonstrated significant intracellular reduction. This aligns with previously published AmB intracellular activity [PMID: 1804000].

Thank you for your valuable inquiries and suggestions, which have greatly strengthened our manuscript. We have diligently incorporated your feedback and hope these revisions persuade you to accept our submission.

Sincerely,

Mohamed Seleem, Ph.D.

Tyler and Frances Young Endowed Chair of Bacteriology

Director, Center for One Health Research, Virginia Tech

1410 Prices Fork RD, Blacksburg, VA, 24061

References
