# Supplementary material for: Repurposing antiviral agents against mucormycosis
Source: PLoS One. 2026 Feb 18;21(2):e0342559. doi: 10.1371/journal.pone.0342559 (PMC12915971; doi:10.1371/journal.pone.0342559)
Supplement: Attachment — Submitted filename: Response to Reviewers 2.docx [file pone.0342559.s005.docx]

Response to Reviewer:

**Reviewer #1: 1. Kindly explain why different subinhbitory concentration of Amphotericin B were used during time kill assays (0.25 microgram / microliter) and screening of agent (0.0625 microgram/microliter) synergy tests**

Response 1:

During the initial screening, a very low concentration of AmB (0.0625 µg/mL) was used to sensitively identify compounds capable of potentiating AmB activity. At this stage, antivirals were tested at a fixed screening concentration of 16 µM, corresponding to approximately 12 µg/mL for daclatasvir and 14 µg/mL for velpatasvir, to maximize hit detection.

In contrast, the time-kill assays were intended to assess the kinetics of antivirals over 48 hours. Therefore, a modestly higher but still sub-inhibitory concentration of AmB (0.25 µg/mL) was used, while the concentrations of daclatasvir and velpatasvir were substantially reduced compared to the screening conditions. As shown in Fig 1 below, AmB at subinhibitory concentrations of 0.0625 or 0.25 µg/mL alone did not significantly inhibit *R. delemar* growth over the assay period. In contrast, combinations containing AmB at 0.25 µg/mL with daclatasvir or velpatasvir (8 µg/mL) resulted in measurable growth suppression for up to 24 h, whereas corresponding combinations using AmB at 0.0625 µg/mL did not produce a comparable effect.

**Figure 1.** Growth kinetics of *Rhizopus delemar* 99-880 treated with AmB alone or in combination with hepatitis C antivirals. **(A)** AmB at subinhibitory concentrations (0.0625 and 0.25 µg/mL) did not significantly inhibit fungal growth. **(B and C)** Combination treatment with AmB (0.25 µg/mL) and DAC (8 µg/mL) or VEL (8 µg/mL) resulted in growth inhibition for up to 24 h, whereas combinations using AmB at 0.0625 µg/mL did not produce sustained inhibition.
